# Diverging trends in the global burden of ischemic heart disease attributable to non-optimal temperatures: a historical analysis (1990–2021) and 2050 projections

**DOI:** 10.3389/fpubh.2025.1593346

**Published:** 2025-07-30

**Authors:** Zehao Jin, Yuting Pang, Ziyang Huang, Jialin Liu, Xiaoyi Zhan, Kangwei Wang

**Affiliations:** ^1^The Affiliated Yueqing Hospital of Wenzhou Medical University, Yueqing, Zhejiang, China; ^2^School of Medicine and Health, Technical University of Munich, Munich, Germany; ^3^The First Affiliated Hospital of Wenzhou Medical University, Wenzhou, Zhejiang, China; ^4^Munich Medical Research School, Lidwig-Maximilians University Munich, Munich, Germany

**Keywords:** non-optimal temperatures, ischemic heart disease, global burden of disease study, mortality, DALYs

## Abstract

**Background:**

This study aimed to comprehensively evaluate the global burden of temperature-related ischemic heart disease from 1990 to 2021, analyzing the temporal trends and regional disparities stratified by socioeconomic development levels. Furthermore, we identified high-risk populations and mapped the trajectory of disease burden up to 2050 to generate data that will inform the establishment of evidence-based public health interventions and climate adaptation strategies.

**Methods:**

A comprehensive analysis was conducted on data derived from the Global Burden of Disease Study 2021 (GBD 2021) to determine the impact of temperature-related ischemic heart disease burden in 204 countries and territories. Primary outcome measures included absolute mortality counts, disability-adjusted life years (DALYs), and age-standardized mortality rates (ASMRs). In addition, temporal trend analysis was conducted using joinpoint regression to identify significant inflection points and calculate the annual percent change (APC) estimates. The future landscape of changes in mortality up to 2050 was predicted using the Bayesian age-period-cohort (BAPC) modeling approach, while accounting for age-specific, period-specific, and birth cohort effects. Socioeconomic stratification was performed using the Sociodemographic Index (SDI) quintiles to compare and characterize the variations in the disease burden across development levels. Data uncertainty was quantified using Monte Carlo simulation methods, and the results were expressed as point estimates and their corresponding 95% uncertainty intervals (UI) to ensure robust statistical inference.

**Results:**

In 2021, high-temperature exposure contributed to 112,389 IHD deaths globally (95% UI: 17,052-256,434), reflecting a 345.0% increase compared to baseline levels in 1990. The corresponding age-standardized mortality rate increased by 1.34 per 10,000, with an estimated annual percentage change (EAPC) of 1.67 (95% CI: 0.61–2.73). The analysis identified marked sex-specific disparities, characterized by a 41.6% (risk ratio: 1.416, 95% CI not provided) higher mortality risk in males relative to females and a male-to-female DALYs ratio of 1.667. In contrast, low non-optimal temperature was associated with 505,298 IHD deaths globally (95% UI: 432,024-619,922), which represented a 64.4% increase in absolute numbers since 1990 (EAPC: 1.09, 95% CI: 0.77–1.40). In contrast, age-standardized mortality rates decreased by 36.9% annually (EAPC: -2.61, 95% CI: −2.73 to −2.48), indicating improved population-level resilience despite the growing absolute burden. Significant socioeconomic disparities were observed, with low-to-middle SDI regions bearing a disproportionate share (75.0%) of the global high non-optimal temperature-related mortality burden. Geographically, North Africa and the Middle East recorded the highest rates (5.97 per 100,000 population), while high-SDI regions demonstrated a sustained annual decline of 6.8% in age-standardized mortality rates linked to low non-optimal temperature. Analysis of the Bayesian modeling projections for 2050 revealed divergent trajectories: high non-optimal temperature-related age-standardized death rates and DALYs rates are likely to increase by 2.85 per 100,000 and 66.83 per 100,000, respectively. In contrast, age-standardized mortality rates associated with low non-optimal temperature are anticipated to decrease by 6.08 per 100,000, reflecting continued adaptation and improved healthcare infrastructure.

**Conclusion:**

Non-optimal temperature exposure exerts differential effects on the global IHD mortality burden. Moreover, disease risks linked to high non-optimal temperatures are exacerbated with anthropogenic climate change, which necessitates the formulation of targeted occupational health interventions and enhanced healthcare infrastructure, particularly in low-resource settings. Conversely, while low non-optimal temperature-related mortality risks exhibited a declining age-standardized rates, the growing absolute burden attributable to population aging and persistent energy inequities underscores the need for continued surveillance and intervention. Finally, the disproportionate effect on socioeconomically disadvantaged regions highlights the urgent need for climate-health equity initiatives.

## 1 Introduction

Increases in the frequency and intensity of non-optimal temperature events due to climate change have emerged as a critical public health challenge. These changes have led to a significant rise in the occurrence of cardiovascular diseases, with IHD being the most prevalent and contributing substantially to the global health burden. Data indicate that from 1990 to 2021, global high non-optimal temperature-related age-standardized ischemia heart disease (IHD) mortality substantially increased by 345.0%, accompanied by a corresponding 322.3% rise in age-standardized disability-adjusted life years (DALYs). While high non-optimal temperature-related mortality has exhibited an alarming trend, low-temperature exposure continues to represent a substantial and persistent threat to cardiovascular health. Current IHD burden attributable to non-optimal temperatures exhibits complex spatial heterogeneity and demographic disparities, driven by the interaction of various factors—including levels of socioeconomic development, population aging dynamics, and disparities in healthcare accessibility across global regions (1, 2).

Existing research demonstrates that prolonged exposure to either high or low non-optimal temperatures exacerbates the risk of developing IHD in multipathway mechanisms. Direct physiological stressors of IHD include altered blood viscosity and endothelial dysfunction, while indirect mechanisms comprise systemic inflammatory responses (3, 4). Outdoor occupational populations—especially those involved in construction and agricultural works—are considered a high-risk population due to sustained environmental exposure (5). Despite these mechanistic insights, comprehensive analyses examining long-term temporal trends and sociodemographic heterogeneity in temperature-attributable IHD burden remain scarce, highlighting the urgent need for targeted, region-specific intervention strategies.

This study uses data from the Global Burden of Disease (GBD) 2021 study integrated with climate exposure metrics, the Socio-Demographic Index (SDI), and Bayesian prediction models to comprehensively evaluate the changing global burden of temperature-attributable IHD. The study aims to elucidate the influence of population aging, economic disparities, and climate adaptation capacity on regional IHD patterns across varying sociodemographic strata, thereby generating robust evidence to guide the development of targeted, climate-responsive public health policy interventions.

## 2 Methods

### 2.1 Data acquisition and sources

This study utilized data from the GBD 2021 study to evaluate the global impact of non-optimal temperature-attributable IHD burden. The GBD 2021 dataset is a publicly available through the Global Health Data Exchange (GHDx) Results Tool (http://ghdx.healthdata.org/), and it provides comprehensive open-access global and regional health metrics. This dataset comprise various estimates for 371 diseases and injuries—including incidence, prevalence, mortality, and DALYs— alongside 88 risk factors spanning behavioral, environmental, occupational, and metabolic domains. In this study, a comprehensive analysis was conducted to examine the burden of IHD attributable to high and low non-optimal temperature exposures across 204 countries and territories from 1990 to 2021. Temperature exposures were categorized based on location-specific percentile thresholds derived from long-term local climate distributions, following the methodology used in the GBD 2021 study and other major multicountry studies (6–9). The SDI was used as the primary measure of socioeconomic development, incorporating total fertility rates, mean years of education, and lag-distributed income per capita. The SDI scale ranges from 0 to 1, with countries stratified into five development tiers: Low, Low-middle, Middle, High-middle, and High SDI regions. This comprehensive approach facilitated systematic trend analysis and in-depth evaluation of temperature-attributable IHD burden across diverse socioeconomic settings. Given that this study used publicly available, de-identified data, ethical approval and informed consent requirements were waived. All analyses were conducted in strict adherence to the established guidelines for accurate and transparent health assessment reporting.

### 2.2 Population and global burden analysis

We analyzed the age-standardized prevalence and mortality of IHD attributable to non-optimal temperature exposure globally, incorporating 95% uncertainty intervals (UIs), using data from the GBD 2021 study, which encompasses 204 countries, 21 GBD regions, and 5 SDI quintiles. The data were stratified by sex and age groups based on the standard GBD age categorization system. High-resolution global maps were used to generate visualizations of the spatial distribution of temperature-attributable IHD burden, highlighting disparities across socio-demographic and geographic settings. This spatial analysis provided critical insights into global patterns of temperature-sensitive cardiovascular disease burden, thereby enabling the identification of regions requiring targeted intervention strategies.

### 2.3 Decomposition analysis

Formal decomposition analysis was conducted to delineate the drivers underlying temporal trends in temperature-attributable IHD burden from 1990 to 2021. This approach deconstructs overall change in disease burden into three principal components: (1) Population growth, which reflecting changes in the absolute number of people; (2) Population aging, encompassing shifts in age distribution toward older, more vulnerable age groups; (3) Epidemiological changes, which involves alterations in age-specific incidence, mortality, and DALY rates due to risk factor transition, clinical interventions, or environmental changes.

Following the established framework of the GBD decomposition methodology, we constructed counterfactual scenarios for each year by systematically holding individual components constant at their baseline (1990) values, thereby isolating their respective contributions to changes in IHD burden. Specifically, for each domain, we quantified the proportional contribution to the overall change in deaths and DALYs by comparing observed values with modeled counterfactuals, thereby enabling robust attribution across time and regions (10–12).

This methodological approach ensures that demographic expansion (population effects), evolving age structure (demographic aging), and shifts in epidemiological risk (age-specific rate changes associated with climate, healthcare, or socioeconomic transitions) are quantitatively distinguished. This approach substantially enhances interpretability and guides targeted public health and policy responses. Our analyses reveal that population growth and demographic aging were the primary contributors to the absolute increase in IHD mortality attributable to both heat and cold exposure. In contrast epidemiological transitions—characterized by improved healthcare access and preventive measures—were associated with declines in age-standardized IHD rates, especially for low non-optimal temperature-related burden across high-SDI regions.

### 2.4 Health inequality analysis

Health inequality analysis was used to systematically examine disparities in disease burden across countries and regions. This analysis provides critical insights to inform evidence-based public health policy development. Two complementary measures were used in the analysis: the Slope Index of Inequality (SII) and the Concentration Index (CI) to quantify health inequalities in temperature-attributable IHD burden. Notably, the SII quantifies the absolute relationship between health outcomes and socioeconomic status through linear regression analysis, with the SDI serving as the primary socioeconomic predictor. This metric quantifies the absolute disparity in health outcomes between the most and least advantaged population groups. The CI, which ranges from −1 to +1, measures the relative distribution of health outcomes across the socioeconomic spectrum. Values approaching zero indicate minimal inequality. Positive values suggest disproportionate burden among lower socioeconomic groups (pro-poor distribution), while negative values indicate a higher burden among wealthier populations (pro-rich distribution). We quantified both the SII and CI for temperature-attributable IHD mortalities and DALYs from 1990 to 2021, evaluating health disparities at global, regional, and national levels across all the 204 countries and territories.

### 2.5 Prediction analysis

The population was stratified by sex and then the Bayesian Age-Period-Cohort (BAPC) models were used to project temperature-attributable IHD trends over 29 years (2022–2050). The insights from this analysis were used to inform policy formulation and resource allocation. The BAPC framework integrates the effects of age, time, and cohort, thereby accounting for changes in demographic and epidemiological patterns. Additionally, we used Integrated Nested Laplace Approximation (INLA) approach integrated with BAPC modeling to efficiently approximate posterior distributions while mitigating convergence issues associated with the traditional Markov Chain Monte Carlo (MCMC) methods. This approach provides for robust quantification of uncertainty and reliable projections of future IHD burden under varying temperature scenarios.

### 2.6 Statistical analysis

All statistical analyses were performed using R (version 4.3.3) and Stata 18 (StataCorp, College Station, TX). Decomposition and sensitivity analyses were performed by developing custom codes, while Bayesian modeling was conducted using WinBUGS (version 1.4). Geographic visualization and spatial analyses were performed using ArcGIS Pro and QGIS (version 3.16) to generate high-resolution maps that illustrate global temperature-attributable IHD burden and regional disparities. Data visualizations, including bilateral and dual-axis plots, were generated using the ‘ggplot2’ and ‘Benchmarking’ packages in R.

### 2.7 Statistical significance

A *p* < 0.05 was considered statistically significant for all analyses, which is consistent with established conventions in epidemiological research and GBD studies. This threshold ensures robust inference while maintaining comparability with existing studies involving analysis of the climate-health relationships.

## 3 Results

### 3.1 Global analysis of temperature-related IHD burden and trends from 1990 to 2021

**Figure 1:**
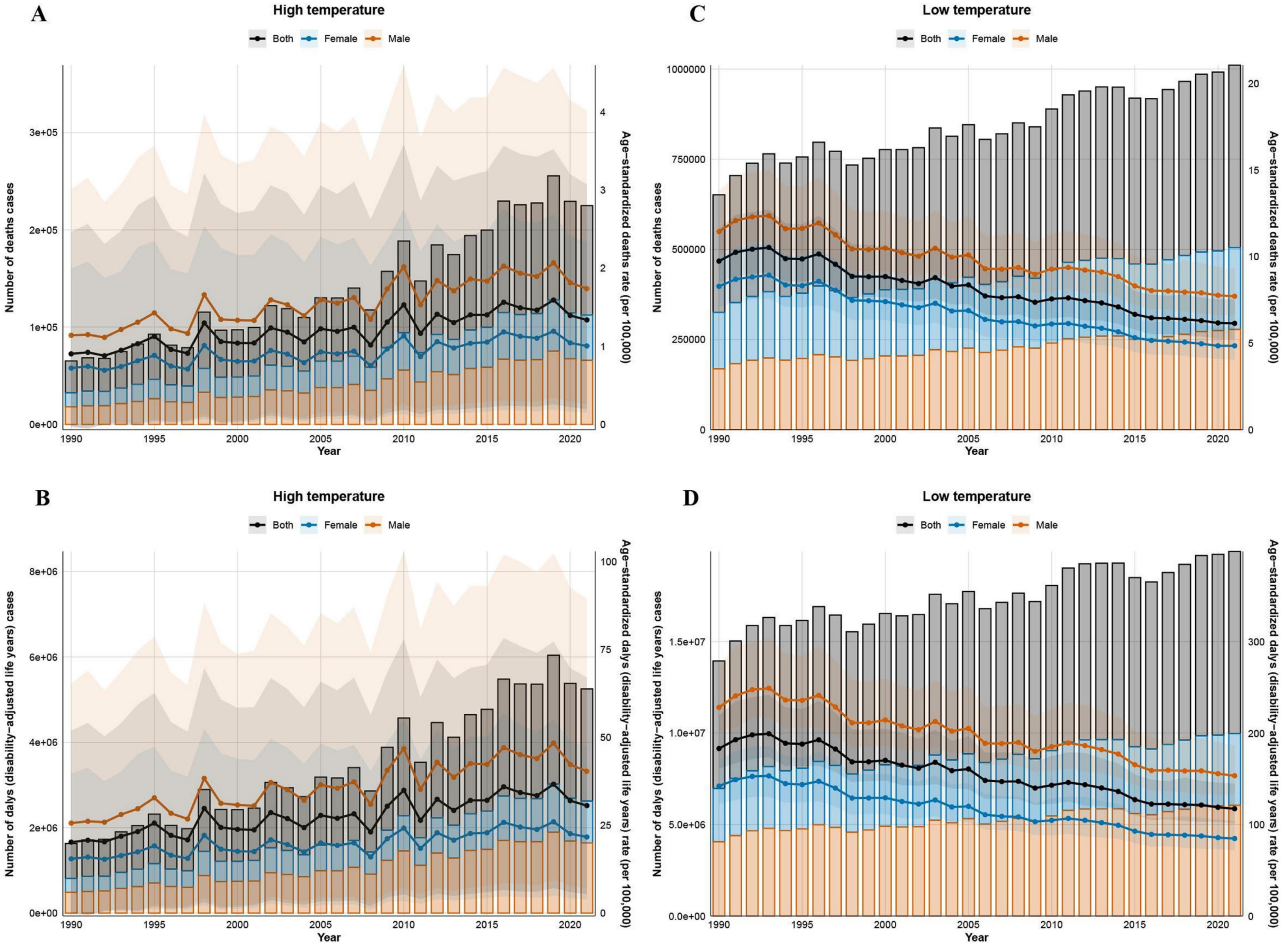
Global trends in the number of deaths, DALYs, and ASR due to ischemic heart disease in high and low non-optimal temperature environments from 1990 to 2021. The number of deaths in high non-optimal temperature environments **(A)**, DALYs in high non-optimal temperature environments **(B)**, deaths in low non-optimal temperature environments **(C)**, and DALYs in low non-optimal temperature environments **(D)**.

Globally, from 1990 to 2021, high non-optimal temperature-attributable IHD burden exhibited significant increases in mortality counts, DALYs, and their associated age-standardized rates (ASRs). Notably, by 2021, high non-optimal temperatures contributed to 112,390 deaths (95% UI: 17,052-256,434) and 2,623,940 DALYs (95% UI: 467,682-5,727,665) globally, with ASRs of 1.34 (95% UI: 0.2–3.07) and 30.57 (95% UI: 5.41–66.961), respectively. From 1990 to 2021, crude IHD death counts and DALYs attributable to non-optimal temperatures surged by 345.0 and 322.3%, respectively. Moreover, ASRs increased by 48.09% (deaths) and 51.49% (DALYs), with estimated annual percentage changes (EAPCs) of 1.67 (95% confidence interval [CI]: 0.52–2.82) and 1.74 (95% CI: 0.67–2.28), respectively. In contrast, low non-optimal temperature-related IHD exhibited a surge in crude death counts and DALYs but declining ASRs. In 2021, low non-optimal temperatures resulted in 505,298 deaths (95% UI: 432,024-619,922) and 9,961,232 DALYs (95% UI: 8,580,363-12,290,759), with ASRs of 6.14 (95% UI: 5.23–7.53) for deaths and 117.40 (95% UI: 101.19–144.86) for DALYs. Compared to 1990, crude death counts and DALYs increased by 55.19 and 42.97%, while ASRs decreased by 36.9 and 35.9%, respectively; additionally, the EAPCs were −1.53 (95% CI: −1.75 to −1.31) for deaths and −1.49 (95% CI: −1.75 to −1.23) for DALYs (Figure 1; Tables 1–4).

**Table 1:** Deaths of low-temperature-related IHD Between 1990 and 2021 at the global and regional level.

Location	Num_1990	ASR_1990	Num_2021	ASR_2021	EAPC_CI
Andean Latin America	864 (701 to 987)	4.7 (3.81 to 5.35)	1,533 (1,215 to 1911)	2.7 (2.15 to 3.37)	−1.81 (−2.89 to −0.72)
Australasia	2,892 (2,585 to 3,369)	12.73 (11.32 to 14.87)	1954 (1,627 to 2,323)	3.18 (2.69 to 3.78)	−4.56 (−4.93 to −4.19)
Caribbean	504 (408 to 628)	2.14 (1.73 to 2.67)	630 (516 to 768)	1.15 (0.94 to 1.41)	−2.48 (−4.37 to −0.56)
Central Asia	10,196 (8,919 to 12,674)	24.8 (21.58 to 30.93)	13,467 (11,649 to 16,634)	20.34 (17.53 to 25.08)	−0.92 (−1.82 to −0.01)
Central Europe	23,034 (20,083 to 27,907)	17.24 (15.03 to 20.94)	22,251 (19,146 to 28,083)	9.4 (8.1 to 11.86)	−2.16 (−2.76 to −1.54)
Central Latin America	3,971 (3,504 to 4,549)	5.62 (4.95 to 6.44)	10,384 (8,974 to 12,421)	4.36 (3.77 to 5.22)	−0.93 (−1.32 to −0.53)
Central Sub-Saharan Africa	476 (336 to 652)	2.77 (1.96 to 3.69)	833 (606 to 1,093)	2 (1.46 to 2.65)	−1.41 (−2.26 to −0.55)
East Asia	42,397 (35,026 to 50,680)	6.97 (5.74 to 8.3)	142,486 (116,459 to 174,315)	7.73 (6.32 to 9.44)	0.37 (−0.61 to 1.37)
Eastern Europe	49,122 (44,549 to 59,468)	20.16 (18.07 to 24.44)	59,135 (50,549 to 75,428)	16.55 (14.14 to 21.11)	−1.04 (−2.51 to 0.46)
Eastern Sub-Saharan Africa	1948 (1,524 to 2,377)	3.04 (2.41 to 3.73)	3,743 (3,048 to 4,615)	2.69 (2.16 to 3.29)	−0.73 (−1.36 to −0.09)
Global	325,588 (285,690 to 387,323)	9.74 (8.52 to 11.6)	505,298 (432,024 to 619,922)	6.14 (5.23 to 7.53)	−1.53 (−1.75 to −1.31)
High-income Asia Pacific	8,925 (7,777 to 9,999)	5.1 (4.38 to 5.74)	11,044 (8,670 to 12,781)	1.83 (1.49 to 2.1)	−3.2 (−3.46 to −2.95)
High-income North America	44,839 (38,555 to 54,406)	12.36 (10.65 to 15.01)	35,619 (29,427 to 42,583)	5.06 (4.24 to 6.04)	−2.99 (−3.49 to −2.49)
High-middle SDI	107,644 (96,963 to 127,764)	13.03 (11.64 to 15.52)	161,776 (138,029 to 198,114)	8.42 (7.17 to 10.32)	−1.58 (−2.32 to −0.83)
High SDI	112,781 (99,382 to 135,438)	10.27 (9 to 12.35)	91,335 (74,987 to 109,635)	3.84 (3.21 to 4.6)	−3.22 (−3.4 to −3.03)
Low-middle SDI	35,692 (26,578 to 45,723)	6.75 (4.99 to 8.67)	81,159 (61,932 to 104,899)	6.24 (4.79 to 8.06)	−0.16 (−0.59 to 0.28)
Low SDI	10,319 (7,942 to 13,190)	5.29 (4.03 to 6.78)	20,462 (15,999 to 25,361)	4.79 (3.74 to 5.92)	−0.3 (−0.84 to 0.24)
Middle SDI	58,688 (48,677 to 70,587)	7.22 (5.96 to 8.63)	150,114 (127,839 to 178,552)	6.44 (5.45 to 7.62)	−0.37 (−0.61 to −0.12)
North Africa and Middle East	28,976 (23,578 to 36,272)	20.81 (16.87 to 26.06)	51,493 (43,318 to 64,601)	13.71 (11.52 to 17.08)	−1.32 (−1.97 to −0.66)
Oceania	123 (93 to 161)	4.69 (3.7 to 6.17)	272 (203 to 360)	4.04 (3.05 to 5.32)	−0.4 (−0.76 to −0.04)
South Asia	35,244 (24,174 to 46,721)	6.86 (4.71 to 9.18)	93,852 (63,020 to 124,192)	6.97 (4.66 to 9.19)	0.21 (−0.25 to 0.67)
Southeast Asia	3,695 (2,827 to 4,801)	1.69 (1.28 to 2.19)	7,504 (5,780 to 9,397)	1.34 (1.02 to 1.68)	−1.13 (−1.96 to −0.3)
Southern Latin America	4,979 (4,580 to 5,628)	11.85 (10.78 to 13.43)	3,507 (3,129 to 3,865)	3.89 (3.48 to 4.28)	−3.38 (−3.68 to −3.08)
Southern Sub-Saharan Africa	1,266 (1,042 to 1,481)	5.38 (4.42 to 6.31)	2,625 (2,318 to 2,992)	5.53 (4.86 to 6.3)	−0.5 (−1.86 to 0.88)
Tropical Latin America	4,255 (3,467 to 5,108)	5.34 (4.28 to 6.41)	5,822 (4,759 to 6,967)	2.31 (1.89 to 2.77)	−3.16 (−4.12 to −2.19)
Western Europe	56,706 (49,248 to 67,644)	9.56 (8.27 to 11.41)	35,646 (28,795 to 42,324)	3.1 (2.57 to 3.65)	−3.69 (−4.09 to −3.29)
Western Sub-Saharan Africa	1,174 (686 to 1736)	1.63 (0.97 to 2.4)	1,497 (969 to 2098)	0.97 (0.64 to 1.35)	−1.8 (−3.78 to 0.22)

**Table 2:** DALYs of Low-temperature-related IHD Between 1990 and 2021 at the global and regional level.

Location	Num_1990	ASR_1990	Num_2021	ASR_2021	EAPC_CI
Andean Latin America	19,310 (15,637 to 22,206)	91.97 (74.41 to 105.45)	30,408 (23,769 to 38,271)	51.29 (40.21 to 64.45)	−1.91 (−3 to −0.8)
Australasia	53,237 (48,335 to 61,920)	229.23 (207.62 to 266.92)	28,978 (24,856 to 34,164)	52.79 (46.04 to 61.92)	−4.79 (−5.23 to −4.34)
Caribbean	10,462 (8,397 to 13,138)	41.03 (33 to 51.5)	12,729 (10,326 to 15,559)	23.63 (19.16 to 28.9)	−2.27 (−3.92 to −0.6)
Central Asia	214,982 (190,233 to 264,927)	474.93 (417.21 to 587.27)	279,830 (241,583 to 346,585)	369.53 (320.13 to 457.3)	−1.13 (−2.21 to −0.05)
Central Europe	461,987 (405,543 to 557,160)	322.86 (282.78 to 389.68)	359,732 (313,184 to 453,549)	160.96 (139.8 to 202.94)	−2.45 (−3.03 to −1.86)
Central Latin America	87,567 (77,146 to 100,384)	106.57 (94.03 to 122.2)	204,492 (177,681 to 245,001)	82.31 (71.58 to 98.63)	−0.97 (−1.41 to −0.52)
Central Sub-Saharan Africa	12,372 (8,663 to 17,197)	57.07 (40.31 to 77.66)	21,326 (15,356 to 28,397)	40.15 (29.22 to 52.74)	−1.5 (−2.35 to −0.64)
East Asia	1,028,789 (848,219 to 1,236,171)	128.67 (106.07 to 153.61)	2,526,438 (2,053,458 to 3,127,920)	126.7 (103.38 to 155.49)	−0.02 (−0.86 to 0.82)
Eastern Europe	974,061 (895,746 to 1,178,547)	369.56 (337.96 to 447.59)	1,056,385 (905,848 to 1,345,127)	303.08 (259.67 to 385.85)	−1.07 (−2.72 to 0.61)
Eastern Sub-Saharan Africa	52,656 (41,251 to 65,888)	67.17 (52.65 to 82.03)	96,833 (78,361 to 120,129)	55.21 (44.94 to 68.16)	−0.98 (−1.65 to −0.31)
Global	6,967,336 (6,137,182 to 8,362,562)	183.27 (161.52 to 219.62)	9,961,231 (8,580,362 to 12,290,759)	117.4 (101.19 to 144.86)	−1.49 (−1.75 to −1.23)
High-income Asia Pacific	162,401 (145,636 to 182,604)	85.55 (75.86 to 95.94)	158,331 (129,990 to 180,678)	33.25 (28.7 to 37.41)	−3 (−3.22 to −2.79)
High-income North America	804,565 (715,054 to 976,708)	230.18 (205.25 to 279.34)	613,910 (535,061 to 730,928)	95.43 (84.48 to 113.16)	−2.92 (−3.35 to −2.49)
High-middle SDI	2,212,526 (2,000,186 to 2,637,370)	237 (213.64 to 281.68)	2,833,786 (2,470,377 to 3,454,141)	146.38 (127.31 to 178.5)	−1.76 (−2.63 to −0.88)
High SDI	2,049,378 (1,840,342 to 2,461,668)	187.15 (168.11 to 224.88)	1,492,427 (1,287,865 to 1,795,630)	72.06 (63.43 to 86.78)	−3.09 (−3.2 to −2.98)
Low-middle SDI	960,195 (715,701 to 1,234,847)	150.2 (111.81 to 192.41)	2,041,109 (1,536,905 to 2,659,163)	137.93 (104.51 to 178.71)	−0.19 (−0.63 to 0.25)
Low SDI	280,661 (215,303 to 356,449)	119.02 (91.62 to 151.98)	525,391 (412,150 to 652,771)	100.4 (78.76 to 124.52)	−0.56 (−1.11 to −0.01)
Middle SDI	1,455,176 (1,197,726 to 1,755,209)	143.32 (118.78 to 172.54)	3,060,593 (2,621,729 to 3,653,430)	119 (101.56 to 142.01)	−0.62 (−0.83 to −0.42)
North Africa and Middle East	725,502 (590,763 to 912,626)	427.36 (347.58 to 535.84)	1,177,135 (982,372 to 1,482,835)	263.22 (221.31 to 331.05)	−1.55 (−2.18 to −0.92)
Oceania	3,747 (2,789 to 5,001)	113.36 (86.71 to 148.65)	8,094 (5,936 to 10,730)	95.73 (71.51 to 126.55)	−0.45 (−0.8 to −0.11)
South Asia	999,393 (685,412 to 1,320,504)	160.64 (110.26 to 212.7)	2,402,862 (1,612,778 to 3,190,395)	157.44 (105.9 to 208.52)	0.07 (−0.4 to 0.53)
Southeast Asia	97,328 (75,283 to 125,256)	36.57 (28.07 to 47.36)	176,159 (136,645 to 218,891)	27.22 (21.09 to 34)	−1.34 (−2.2 to −0.47)
Southern Latin America	99,164 (92,322 to 111,694)	221.26 (205.66 to 249.58)	64,804 (59,115 to 70,976)	74.54 (68.16 to 81.6)	−3.33 (−3.56 to −3.11)
Southern Sub-Saharan Africa	31,276 (26,559 to 36,226)	112.08 (92.72 to 130.59)	61,309 (53,692 to 69,573)	108.45 (95.74 to 123.45)	−0.71 (−2.07 to 0.68)
Tropical Latin America	104,401 (85,279 to 124,760)	112.87 (92.02 to 135.37)	131,529 (107,967 to 156,865)	50.86 (41.78 to 60.69)	−3.1 (−4.13 to −2.05)
Western Europe	997,109 (880,934 to 1,188,905)	173.43 (153.18 to 206.66)	514,756 (434,634 to 604,500)	52.42 (45.15 to 61.12)	−3.88 (−4.22 to −3.55)
Western Sub-Saharan Africa	27,027 (15,362 to 40,276)	32.03 (18.52 to 47.54)	35,191 (22,145 to 49,557)	18.49 (11.94 to 25.92)	−1.9 (−3.93 to 0.16)

**Table 3:** Deaths of high-temperature-related IHD Between 1990 and 2021 at the global and regional level.

Location	Num_1990	ASR_1990	Num_2021	ASR_2021	EAPC_CI
Andean Latin America	−14 (−34 to −4)	−0.08 (−0.18 to −0.02)	−1 (−33 to 33)	0 (−0.06 to 0.06)	--
Australasia	56 (−26 to 229)	0.25 (−0.11 to 1.01)	23 (−32 to 116)	0.04 (−0.05 to 0.19)	−4.27(−7.17 to −1.29)
Caribbean	2 (−117 to 57)	0.02 (−0.46 to 0.24)	51 (−21 to 119)	0.09 (−0.04 to 0.22)	--
Central Asia	833 (−36 to 2,818)	2.03 (−0.09 to 6.88)	2,287 (341 to 6,452)	3.44 (0.51 to 9.65)	1.2(0.12 to 2.29)
Central Europe	623 (−189 to 2,679)	0.46 (−0.14 to 2)	1,282 (−95 to 4,866)	0.54 (−0.04 to 2.06)	0(−1.73 to 1.76)
Central Latin America	−147 (−497 to 120)	−0.21 (−0.7 to 0.16)	840 (−17 to 1747)	0.35 (−0.01 to 0.73)	--
Central Sub-Saharan Africa	−129 (−597 to −20)	−0.74 (−3.42 to −0.12)	−111 (−377 to 21)	−0.26 (−0.9 to 0.04)	--
East Asia	3,077 (−2,319 to 11,683)	0.52 (−0.39 to 1.96)	15,490 (−6,011 to 56,321)	0.85 (−0.33 to 3.08)	1.66(0.52 to 2.82)
Eastern Europe	765 (−167 to 3,233)	0.31 (−0.07 to 1.33)	2,802 (109 to 10,311)	0.78 (0.03 to 2.88)	2.47(−1.86 to 6.99)
Eastern Sub-Saharan Africa	9 (−178 to 149)	0.01 (−0.3 to 0.23)	149 (−184 to 603)	0.1 (−0.12 to 0.41)	
Global	32,569 (−675 to 86,633)	0.9 (−0.03 to 2.46)	112,390 (17,052 to 256,434)	1.34 (0.2 to 3.07)	1.67(0.61 to 2.73)
High-income Asia Pacific	558 (−207 to 2,112)	0.32 (−0.12 to 1.2)	647 (−206 to 2,520)	0.11 (−0.03 to 0.42)	−2.84(−4.18 to −1.49)
High-income North America	3,390 (−625 to 12,739)	0.93 (−0.17 to 3.51)	3,523 (−877 to 12,963)	0.5 (−0.13 to 1.84)	−1.86(−2.53 to −1.2)
High-middle SDI	3,688 (−1,506 to 14,669)	0.44 (−0.18 to 1.76)	13,445 (−3,337 to 46,855)	0.7 (−0.18 to 2.45)	--
High SDI	5,710 (−843 to 20,607)	0.53 (−0.08 to 1.89)	9,186 (−482 to 28,525)	0.45 (0 to 1.31)	−0.32(−0.81 to 0.18)
Low-middle SDI	13,156 (1730 to 25,329)	2.42 (0.29 to 4.71)	45,828 (12,527 to 86,439)	3.49 (0.96 to 6.57)	2.12(0.71 to 3.56)
Low SDI	2,158 (−445 to 5,090)	1.13 (−0.26 to 2.68)	7,218 (1,077 to 14,336)	1.71 (0.26 to 3.36)	1.37(−0.44 to 3.21)
Middle SDI	7,842 (−948 to 19,930)	0.92 (−0.13 to 2.4)	36,664 (6,113 to 82,777)	1.51 (0.24 to 3.47)	2.01(0.93 to 3.11)
North Africa and Middle East	6,940 (519 to 16,247)	4.87 (0.33 to 11.48)	23,738 (4,474 to 51,852)	5.97 (1.09 to 13.07)	0.51(0.06 to 0.97)
Oceania	−4 (−16 to −1)	−0.17 (−0.64 to −0.03)	−3 (−18 to 3)	−0.04 (−0.28 to 0.05)	--
South Asia	14,821 (2,583 to 27,356)	2.91 (0.47 to 5.41)	53,579 (15,454 to 95,187)	3.98 (1.16 to 7.06)	1.68(0.17 to 3.21)
Southeast Asia	193 (−1,613 to 1,241)	0.09 (−0.69 to 0.59)	4,279 (2073 to 6,804)	0.78 (0.36 to 1.26)	12.37(−1.56 to 28.28)
Southern Latin America	94 (−92 to 399)	0.22 (−0.22 to 0.95)	64 (−104 to 317)	0.07 (−0.12 to 0.35)	−2.38(−4.73 to 0.03)
Southern Sub-Saharan Africa	3 (−40 to 59)	0.01 (−0.17 to 0.25)	18 (−102 to 167)	0.04 (−0.21 to 0.34)	--
Tropical Latin America	−144 (−526 to 325)	−0.18 (−0.66 to 0.42)	−64 (−549 to 577)	−0.02 (−0.22 to 0.23)	--
Western Europe	899 (−266 to 4,186)	0.15 (−0.04 to 0.71)	877 (−222 to 3,984)	0.08 (−0.02 to 0.34)	−1.61(−2.69 to −0.51)
Western Sub-Saharan Africa	744 (−739 to 2003)	1.01 (−1.02 to 2.71)	2,919 (700 to 5,286)	1.89 (0.48 to 3.42)	2.22(0.73 to 3.74)

**Table 4:** DALYs of High-temperature-related IHD between 1990 and 2021 at the global and regional level.

Location	Num_1990	ASR_1990	Num_2021	ASR_2021	EAPC_CI
Andean Latin America	−314 (−743 to −77)	−1.5 (−3.55 to −0.37)	−14 (−662 to 680)	−0.02 (−1.12 to 1.15)	--
Australasia	1,027 (−485 to 4,170)	4.43 (−2.08 to 17.99)	344 (−479 to 1,695)	0.63 (−0.88 to 3.07)	−4.5(−7.48 to −1.42)
Caribbean	−182 (−3,043 to 1,126)	−0.53 (−11.31 to 4.45)	725 (−890 to 2,201)	1.31 (−1.69 to 4.06)	--
Central Asia	17,574 (−790 to 59,157)	38.82 (−1.73 to 130.92)	48,259 (7,492 to 136,743)	63.34 (9.67 to 178.9)	1.02(−0.22 to 2.29)
Central Europe	12,393 (−3,753 to 53,299)	8.65 (−2.62 to 37.12)	20,935 (−1,577 to 80,275)	9.4 (−0.72 to 36.14)	−0.26(−2.03 to 1.54)
Central Latin America	−3,269 (−11,298 to 2,711)	−3.99 (−13.6 to 3.24)	17,472 (−496 to 36,493)	6.98 (−0.19 to 14.58)	--
Central Sub-Saharan Africa	−3,385 (−15,488 to −506)	−15.47 (−71.15 to −2.39)	−2,866 (−9,799 to 605)	−5.35 (−18.16 to 0.99)	--
East Asia	73,333 (−55,420 to 278,323)	9.34 (−7.01 to 35.46)	272,574 (−107,898 to 981,524)	13.75 (−5.48 to 49.9)	1.31(0.34 to 2.29)
Eastern Europe	14,815 (−3,485 to 62,987)	5.62 (−1.32 to 23.9)	49,273 (1711 to 182,205)	14.08 (0.48 to 52.05)	2.44(−2.05 to 7.13)
Eastern Sub-Saharan Africa	326 (−4,794 to 4,151)	0.33 (−6.17 to 5.2)	4,133 (−5,042 to 16,564)	2.24 (−2.73 to 8.99)	--
Global	813,984 (3,056 to 2,042,308)	20.18 (−0.12 to 51.93)	2,623,940 (467,681 to 5,727,664)	30.57 (5.41 to 66.96)	1.74(0.67 to 2.82)
High-income Asia Pacific	10,328 (−4,031 to 39,079)	5.42 (−2.09 to 20.55)	9,577 (−2,949 to 36,612)	2.08 (−0.64 to 7.8)	−2.58(−3.88 to −1.27)
High-income North America	60,811 (−11,415 to 227,500)	17.4 (−3.28 to 65.05)	60,609 (−15,512 to 221,956)	9.41 (−2.44 to 34.4)	−1.79(−2.45 to −1.13)
High-middle SDI	80,526 (−33,463 to 310,966)	8.46 (−3.53 to 33.02)	243,057 (−57,272 to 828,097)	12.67 (−3.03 to 43.09)	1.12(−0.73 to 3)
High SDI	110,946 (−13,041 to 386,360)	10.29 (−1.15 to 35.64)	206,268 (9,553 to 570,322)	12.05 (0.95 to 31.71)	0.67(0.18 to 1.17)
Low-middle SDI	359,615 (49,161 to 690,157)	55.56 (7.38 to 106.82)	1,169,779 (314,977 to 2,214,820)	78.49 (21.27 to 148.31)	1.61(0.44 to 2.81)
Low SDI	57,961 (−11,493 to 135,912)	24.9 (−5.09 to 58.76)	182,248 (25,887 to 367,294)	35.29 (5.17 to 70.32)	1.86(0.51 to 3.24)
Middle SDI	204,634 (−19,098 to 507,057)	19.46 (−2.17 to 49.1)	821,556 (156,803 to 1,786,205)	31.05 (5.67 to 68.28)	1.91(0.8 to 3.02)
North Africa and Middle East	177,222 (14,170 to 413,728)	103 (7.94 to 240.92)	593,011 (113,901 to 1,286,131)	125.79 (23.92 to 274)	0.52(0.02 to 1.02)
Oceania	−121 (−487 to −19)	−3.82 (−14.97 to −0.62)	−78 (−539 to 91)	−0.94 (−6.44 to 1.09)	--
South Asia	418,181 (74,860 to 768,270)	67.51 (11.81 to 124.51)	1,371,636 (386,200 to 2,455,184)	89.91 (25.58 to 160.49)	1.55(0.04 to 3.09)
Southeast Asia	4,168 (−46,270 to 30,676)	1.79 (−16.45 to 12.1)	98,213 (50,485 to 153,201)	15.37 (7.73 to 24.22)	12(−0.52 to 26.1)
Southern Latin America	1890 (−1847 to 7,978)	4.22 (−4.12 to 17.8)	1,169 (−1934 to 5,818)	1.34 (−2.23 to 6.68)	−2.39(−4.66 to −0.06)
Southern Sub-Saharan Africa	64 (−977 to 1,426)	0.25 (−3.53 to 5.18)	438 (−2,485 to 4,073)	0.75 (−4.27 to 7.01)	--
Tropical Latin America	−3,686 (−12,982 to 7,797)	−3.87 (−13.95 to 8.56)	−1,629 (−12,518 to 12,865)	−0.62 (−4.84 to 4.98)	--
Western Europe	15,685 (−4,881 to 72,920)	2.73 (−0.86 to 12.69)	12,519 (−3,182 to 56,252)	1.27 (−0.33 to 5.66)	−1.83(−2.9 to −0.76)
Western Sub-Saharan Africa	17,122 (−18,523 to 47,681)	20.19 (−21.05 to 55.24)	67,640 (14,806 to 123,077)	36.01 (8.49 to 65.21)	2.09(0.6 to 3.59)

### 3.2 Subgroup analysis of temperature-related IHD burden stratified by sex and age

**Figure 2:**
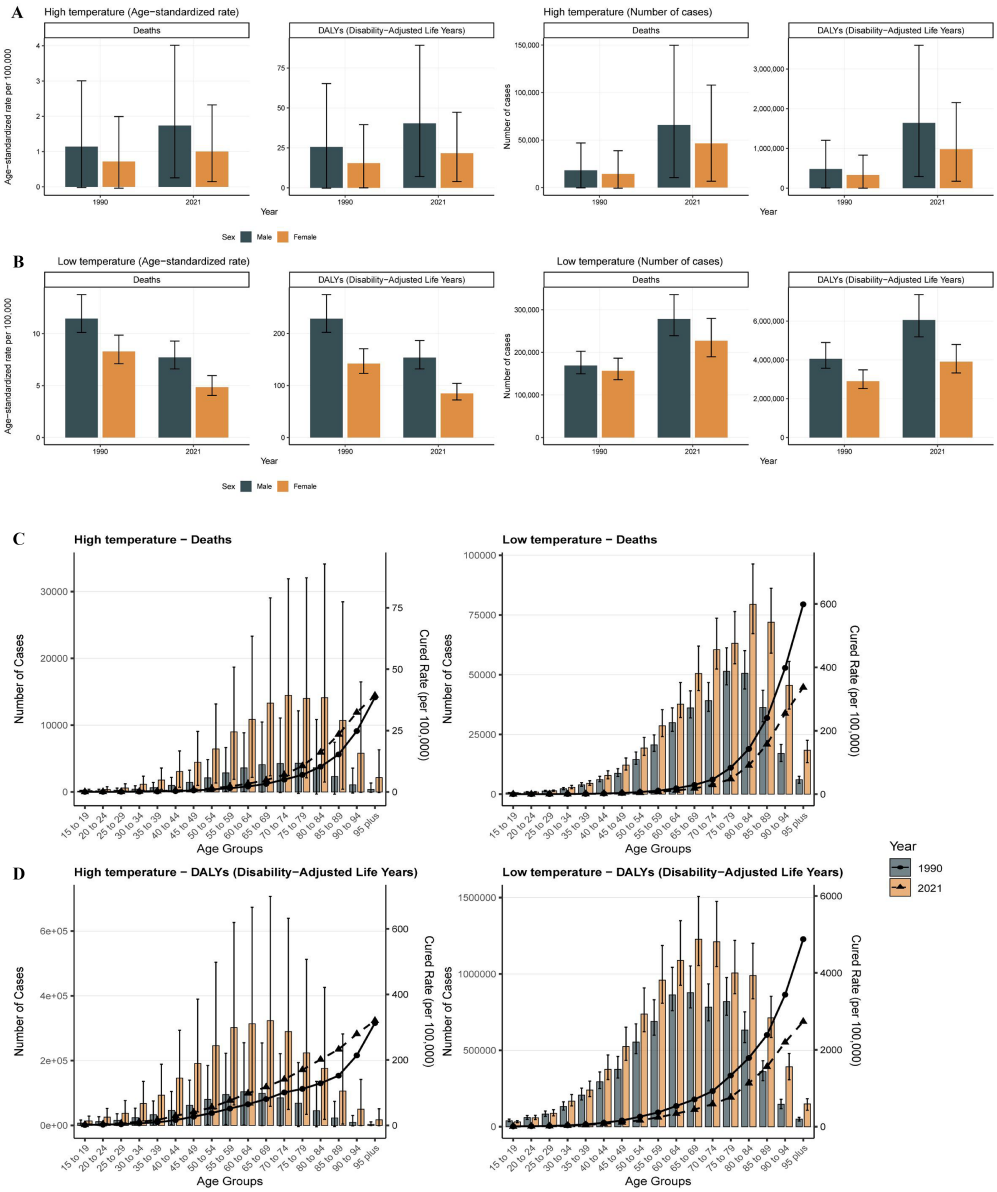
The trends in the number of deaths, DALYs, and ASR for patients with ischemic heart disease stratified by gender and age in high and low non-optimal temperature environments from 1990 to 2021. The number of deaths for different genders in high-temperature environments DALYs **(A)**, DALYs in high non-optimal temperature environment **(B)**, number of deaths in low non-optimal temperature environment **(C)**, DALYs in low non-optimal temperature environment **(D)**.

Throughout the study period from 1990 to 2021, males consistently demonstrated higher temperature attributable IHD burden compared to their female counterparts across all metrics. Notably, a progressively expanding sex disparities were observed under both temperature extremes. For high non-optimal temperature-related IHD, from 1990 to 2021, male-to-female ratios exhibited a significant increase: death ratios increased from 1.263:1 to 1.416:1, while DALY ratios increased from 1.466:1 to 1.667:1. Similarly, low non-optimal temperature-related IHD showed expanding sex disparities, with death ratios increasing from 1.077:1 to 1.224:1 and DALY ratios rising from 1.396:1 to 1.549:1 over the 30-year period (Figure 2).

Age-stratified analysis revealed distinct temperature-specific mortality patterns with pronounced risks associated with age. The ASRs demonstrated consistent stepwise increases with advancing age, with deaths among individuals aged 15–74 years showing a steady increase from 1990 to 2021. However, temperature-specific mortality patterns diverged in older populations: high non-optimal temperature-related deaths plateaued at ages 70–74 years before declining after age 85 years, while low non-optimal temperature-related deaths peaked at ages 80–85 years before exhibiting a subsequent decline. Notably, the DALY patterns showed earlier peak burden, with maximum values occurring at ages 65–69 years across both temperature extremes, followed by a gradual decline in individuals within the advanced age groups. The magnitude of ASR increases demonstrated a strong positive correlation with age, with the most pronounced burden escalations observed in populations at advanced ages, indicating increases physiological vulnerability to temperature extremes (Figure 2).

### 3.3 Subgroup analysis of temperature-related IHD burden stratified by SDI, region, and country

**Figure 3:**
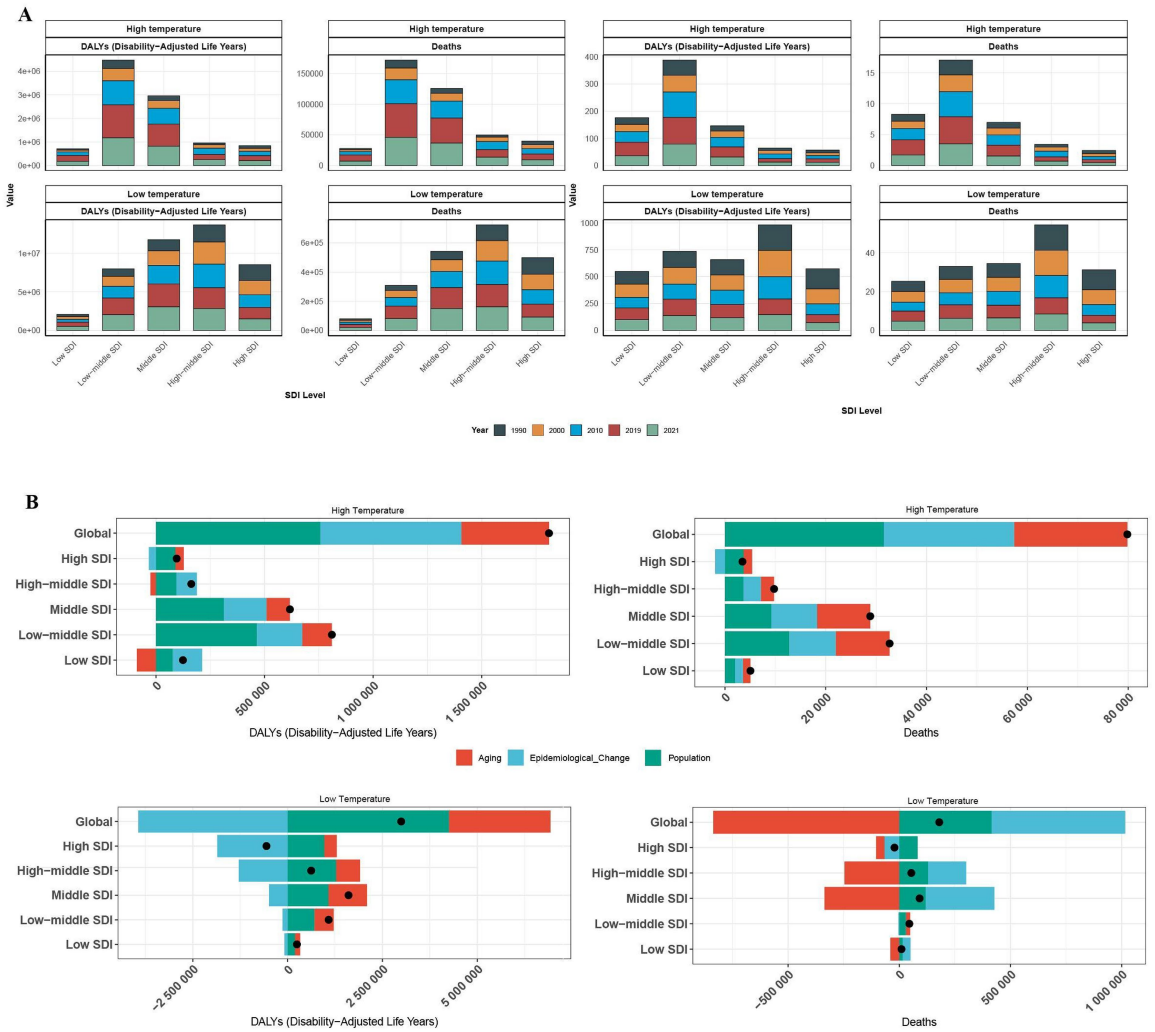
The number of deaths, DALYs, and the proportional contribution of risk factors among patients with ischemic heart disease across varying SDI levels in both high and low non-optimal temperature environments. The death toll of varying SDI levels in both high and low non-optimal temperature environments across varying years for DALYs **(A)**. The proportional contribution of risk factors associated with death and DALYs among varying SDI levels **(B)**.

From 1990 to 2019, SDI-stratified analysis revealed that high non-optimal temperature-related IHD deaths and ASRs exhibited sustained increases across all regions, with a notable decline after 2019. This post-2019 trend reversal likely reflects a confluence of COVID-19-associated cardiovascular complications and intensified global public health interventions implemented during the pandemic period. The global burden distribution exhibited significant socioeconomic disparities, with middle and low-middle SDI regions accounting for 75.0% of high non-optimal temperature-related deaths and 77.4% of DALYs. The Low-middle SDI countries showed disproportionately elevated contributions, underscoring the substantial strain within the healthcare system in regions with limited healthcare resources. Furthermore, ASR analysis confirmed a significant inverse relationship between SDI levels and proportional disease burden. Notably, despite the lower-income regions contributing the least to global greenhouse gas emissions, they experienced the highest temperature-attributable IHD rates (Figure 3).

Analyses of cold exposure revealed increasing deaths and DALYs across all the SDI quintiles, except for the high-SDI regions, where protective trends were observed. Middle-high and middle-SDI regions collectively accounted for 60.1% of global low non-optimal temperature-related deaths and 57.5% of DALYs. Analysis of ASRs underscored the heightened vulnerability of middle-high SDI regions, primarily due to cold-related cardiovascular impacts compounded by aging populations, disparities in healthcare resources, and inadequate climate adaptation infrastructure (Figure 3).

Decomposition analyses revealed substantial heterogeneity in the underlying drivers of global IHD burden attributable to heat stress conditions. Globally, population growth emerged as the predominant contributor, accounting for 39.54% of deaths and 41.82% of DALYs, followed by epidemiological changes contributing to 32.34% of deaths and 35.86% of DALYs. Notably, population aging contributed minimally to the overall trajectory of the IHD burden. However, these driving forces exhibited substantial variation across SDI strata, reflecting complex and context-specific socioeconomic patterns. In high-SDI regions, population dynamics persisted as the primary drivers of deaths despite diminished epidemiological influences and amplified aging effects. Conversely, as SDI levels decreased, epidemiological transitions gained increasing prominence while population factors persisted as dominant contributors, resulting in a pattern that correlated strongly with escalating mortality rates in lower-resource settings. The DALY burden exhibited distinct patterns: population-driven impacts declined progressively with rising SDI levels, whereas the influence of population aging became more pronounced in higher-SDI regions. The contribution of epidemiological transitions varied across the development spectrum, peaking in middle and middle-high-SDI regions before declining in the highest SDI tier.

Under cold exposure conditions, epidemiological transitions emerged as the predominant determinant of global mortality burden, while demographic expansion was the primary driver of DALYs. Remarkably, population aging exhibited a paradoxical protective effect on cold-associated mortality, while epidemiological transitions contributed to reductions in cold-attributable DALYs by enhancing health system resilience and preventive care. The SDI-stratified analyses revealed significant regional heterogeneity: aging consistently emerged as a protective determinant against mortality across high-, high-middle-, middle-, and low-SDI regions, whereas epidemiological transitions exhibited protective effects, specifically within the low-middle-SDI territories. For the DALYs, epidemiological transitions universally demonstrated protective associations across all SDI strata, while population size remained the predominant contributing factor to the overall burden. These epidemiological patterns underscore fundamental underlying disparities in occupational health protections, healthcare system accessibility, and climate adaptation capacity among nations at varying stages of development.

**Figure 4:**
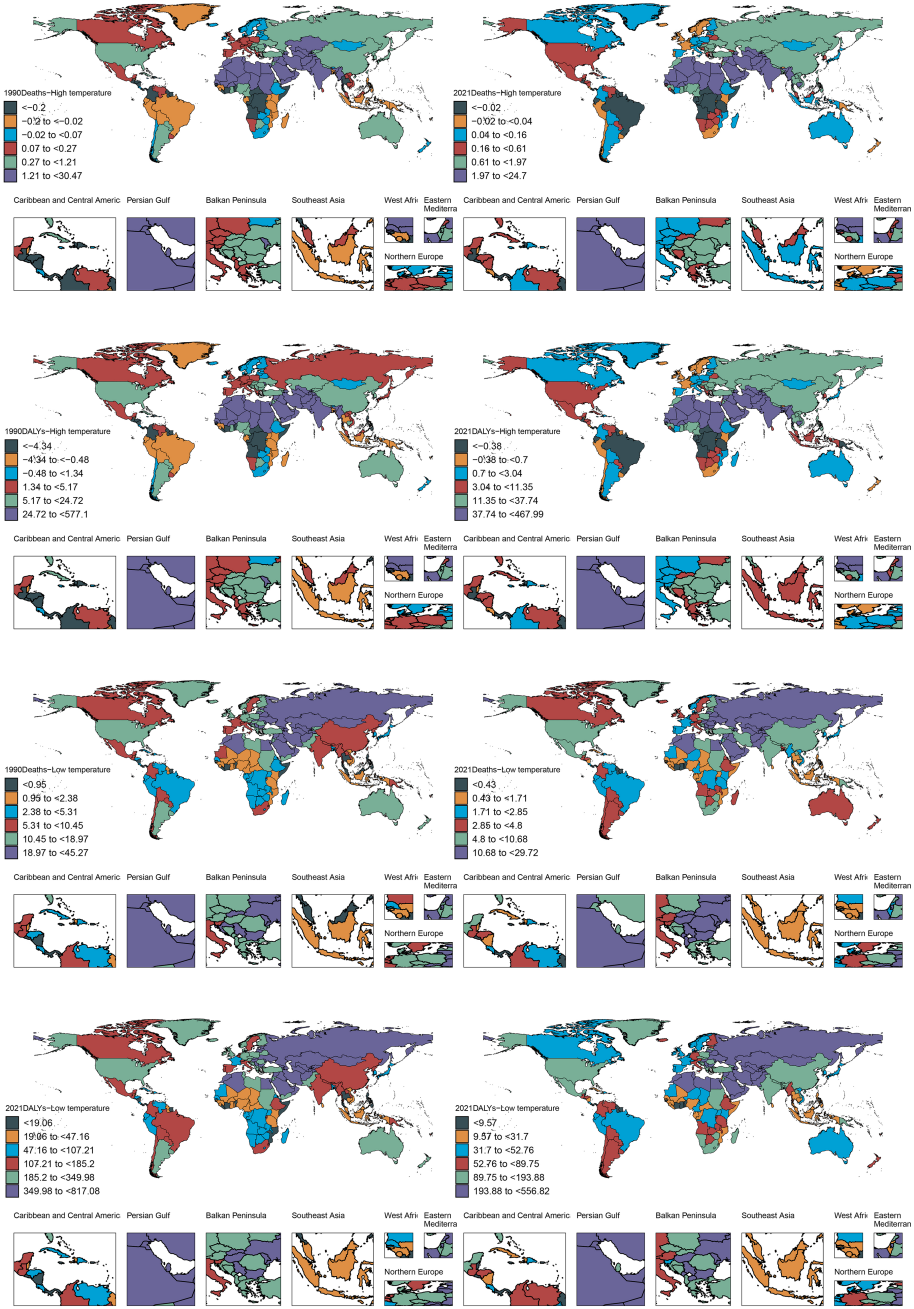
Between 1990 and 2021, the number of deaths associated with patients with ischemic heart disease in both high and low non-optimal temperature environments in varying regions and countries globally for DALYs.

The GBD regional analysis revealed profound geographical heterogeneity in heat-attributable IHD burden in 2021. South Asia demonstrated the most substantial disease burden, accounting for 53,579 deaths (95% UI: 15,454-95,187) and 1,371,636 DALYs (95% UI: 386,200 - 2,455,184). Geographic clustering of elevated age-standardized rates (ASR) was predominantly observed in North Africa and Middle East, Central Asia, Western Sub-Saharan Africa, and East Asia regions. Temporal trend analysis spanning 1990 to 2021 revealed varying epidemiological trajectories characterized by distinct socio-demographic stratification. Low-SDI regions, particularly South Asia and North Africa & Middle East, exhibited increasing EAPC in ASR metrics—deaths EAPC of 0.51 and DALYs EAPC of 0.52—contrasting markedly with pronounced declining trends observed in high-SDI territories. Australasia demonstrated the most substantial reductions in disease burden, with deaths and DALYs EAPC values of −4.27 (95% CI: −7.17 to −1.29) and −6.4 (95% CI: −7.48 to −1.42), respectively. Similarly, Western Europe exhibited comparable declining patterns at −1.61 (95% CI: −2.69 to −0.51) for deaths and −1.83 (95% CI: −2.9 to −0.76) for DALYs. On the other hand, high-middle SDI regions exhibited intermediate reductions in disease burden, for instance, East Asia demonstrated deaths EAPC of −4.73 (95% CI: −5.1 to −4.36) (Figure 4).

**Figure 5:**
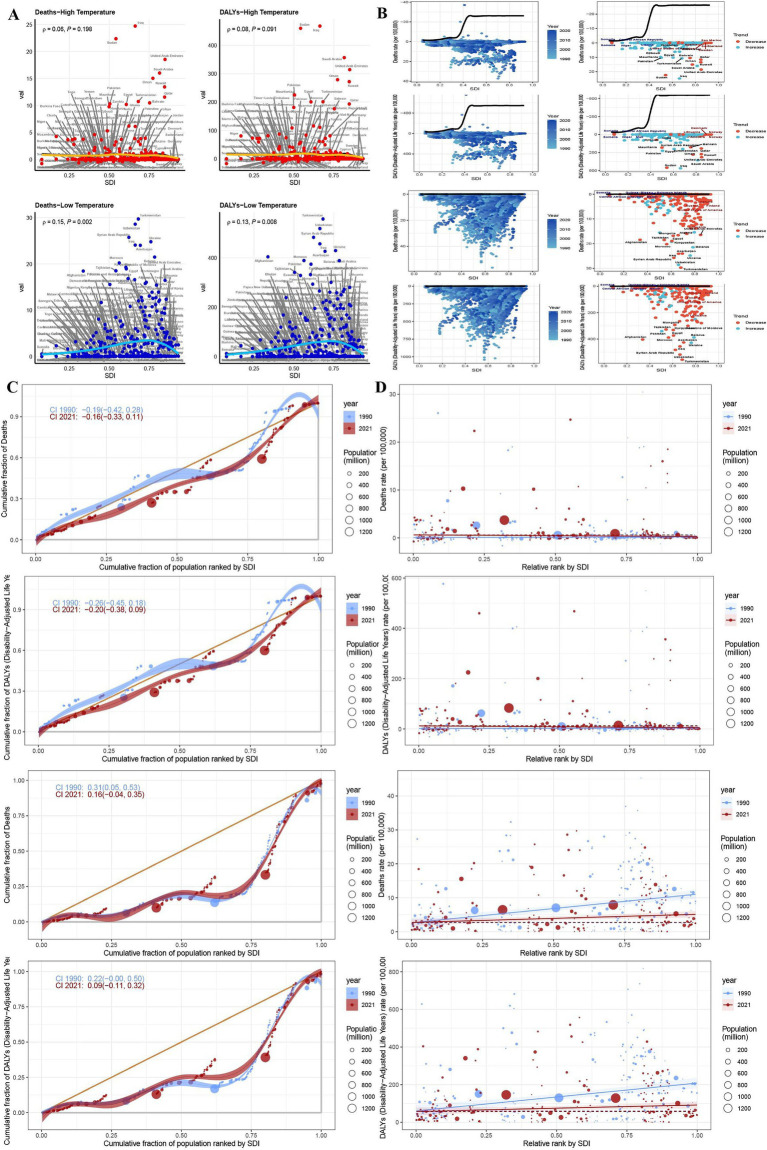
Environments and trends in health inequality index between 1990 and 2021. Regional correlation between deaths and DALYs in patients with ischemic heart disease in both high and low non-optimal temperature environments **(A)**. Frontier analysis of deaths and DALYs in patients with ischemic heart disease in both high and low non-optimal temperature environments in different countries **(B)**. Trends in changes in health inequality index in both high and low non-optimal temperature environments in different countries **(C,D)**.

Nation-level data from the GBD study identified India, China, Pakistan, and Egypt as the principal contributors to global heat-attributable IHD mortality and morbidity burden in 2021. Notably, Pakistan surpassed China in terms of DALY rankings, securing the second-highest global position. India demonstrated a marked predominance, accounting for 40,245 deaths (95% CI: 13,140–68,121), which represents an 8.5-fold higher mortality burden compared to Egypt, the fourth-ranked country. Furthermore, mortality toll and DALYs of India exceeded those of China, the second-ranking country, by factors of 2.65 and 3.83, respectively. This marked disparity presumably reflects the heightened frequency of extreme heat events and chronic summer electrical grid insufficiency in India, which collectively exacerbates the risk of experiencing cardiovascular events. Based on the ASR analysis, Iraq, Sudan, the United Arab Emirates, and Saudi Arabia consistently occupied the top quartile globally for both mortality and DALYs. Iraq reported 24,704 deaths (95% CI: 4,588-47,069) and 467,989 DALYs (95% CI: 86,788-900,066), while Sudan registered 22,642 deaths (95% CI: 2,374-41,049) with substantially elevated DALYs reaching 460,015 (95% CI: 225,440-815,726). Notably, these countries are geographically situated within arid and semi-arid zones of the Middle East and North Africa (MENA) region, sharing three convergent epidemiological determinants: (1) sustained summer ambient temperatures exceeding 45°C; (2) substantial economic dependence on outdoor occupational activities; (3) comparatively limited capacity in healthcare infrastructure. The synergistic interaction between this distinctive geoclimate-occupational exposure nexus may substantially compromise clinical outcomes and long-term survival among individuals with IHD (Figure 5).

Concentration index analysis demonstrated that both mortality and DALYs attributable to IHD in 2021 exhibited increased upward trajectories compared to those in the 1990 baseline values. Notably, the magnitude of mortality increase exhibited an inverse relationship with SDI stratification, with higher-SDI regions demonstrating attenuated incremental burden. Slope index analysis revealed a temporal decline in overall mortality trends from −0.19 (95% CI: −0.42 to 0.28) in 1990 to −0.16 (95% CI: −0.33 to 0.11) in 2021, while the corresponding DALYs index decreased from −0.26 (95% CI: −0.45 to 0.18) to −0.20 (95% CI: −0.38 to 0.09) based on combined slope index methodology. China and India, as the two most populous nations, substantially exceeded the mean population-adjusted burden thresholds for both mortality and DALYs. The mortality burden of China significantly increased from 2,996 deaths (95% CI: −2,216 to 11,241) in 1990 to 15,144 (95% CI: −5,763 to 54,802) in 2021. Moreover, India demonstrated an even more pronounced increase with 10,874 deaths (95% CI: 2,320 to 19,061) in 1990 and 40,245 deaths (95% CI: 13,140 to 68,121) in 2021. The corresponding DALYs increased by 3.715-fold and 3.192-fold for China and India, respectively. These epidemiological patterns suggest an accelerated disease burden trajectory, potentially attributable to healthcare resource inequities, demographic transition dynamics, and intensified environmental exposure (Figure 3).

Substantial geographical heterogeneity in IHD management efficacy was reported in 2021. Sudan and Iraq reported the most suboptimal clinical outcomes, with Sudan recording 24.704 deaths (eff-diff = 52.269) and 467 DALYs (eff-diff = 1,061.954), indicating deteriorating epidemiological trends. Similarly, Iraq reported 22.355 deaths (eff-diff = 48.111) and 460.015 DALYs (eff-diff = 1,014.805) with declining trajectories, presumably attributable to collapse in healthcare infrastructure due to protracted geopolitical conflicts. In contrast, both Somalia (−0.774, eff-diff = 0.878; −17.036, eff-diff = 19.188) and the Central African Republic (−0.641, eff-diff = 3.494; −13.703, eff-diff = 85.706) demonstrated optimal outcomes in the disease management. Cross-national disparities in IHD burden and therapeutic efficacy were initially amplified across lower SDI strata, however this was diminished SDI values above the 0.7 threshold, resulting in the formation of a characteristic U-shaped epidemiological distribution.

Under cold exposure conditions, East Asia demonstrated the highest mortality and DALY burden in 2021, followed by South Asia and Eastern Europe. The ASRs peaked in Eastern Europe for mortality outcomes and Central Europe for DALYs. Globally, China, India, Russia, and the United States exhibited the highest absolute mortality burden. China’s mortality and DALY metrics reached 139,587 deaths (95% CI: 113,521-170,845) and 2,464,745 DALYs (95% CI: 1,996,480-3,056,584), respectively, with the DALYs exceeding those of the second-ranked Democratic People’s Republic of Korea by 49-fold. This disproportionate burden is potentially attributable to China’s large population size, advanced stage of demographic transition, and exposure to severe winter climatic conditions.

The highest ASRs for mortality and morbidity metrics were registered by Turkmenistan, Uzbekistan, and Syria, with Turkmenistan recording 556.818 deaths per 100,000 population (95% CI: 433.957–734.690) and Uzbekistan reporting 518.094 deaths per 100,000 population (95% CI: 432.158–652.214). Although demographically populous nations exhibited elevated absolute burden values due to population scale effects, their corresponding ASRs remained below the highest tertile, suggesting partial risk mitigation through healthcare system interventions. Notably, systemic socioeconomic inequities—including disparities in energy access, geopolitical instability, and inadequate occupational safety frameworks—compounded by cold exposure vulnerability and extreme meteorological conditions, critically compromised IHD clinical outcomes in Turkmenistan, Uzbekistan, and Syria (Figure 5).

### 3.4 Predictive analysis

**Figure 6:**
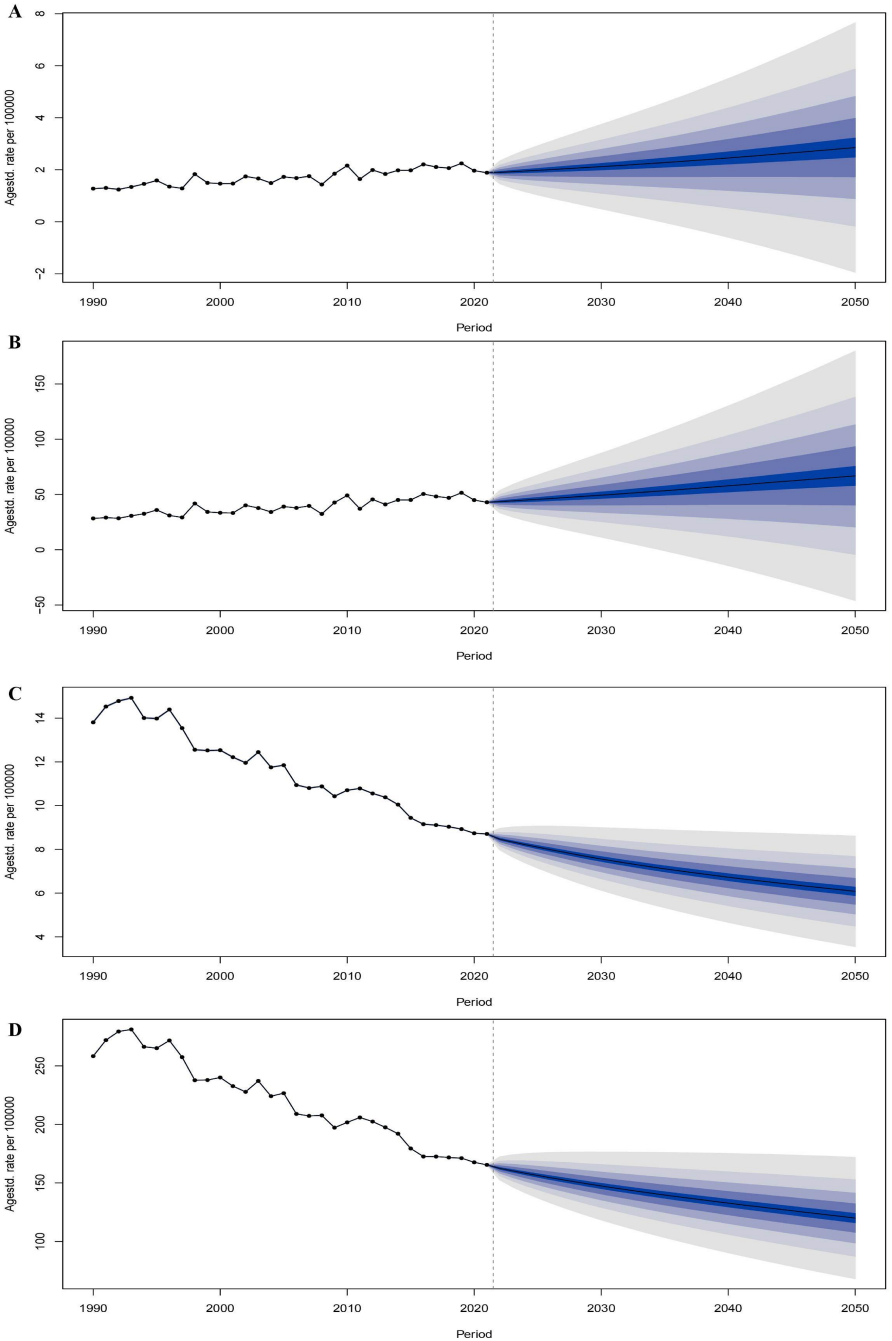
Trends and predictions in the number of deaths and DALYs of patients with ischemic heart disease globally in both high and low non-optimal temperature environments from 1990 to 2050. The trend and prediction of the number of deaths in patients with ischemic heart disease in both high non-optimal temperature environments **(A)**. The trend and prediction of DALYs in patients with ischemic heart disease in both high non-optimal temperature environments **(B)**. The trend and prediction of the number of deaths in patients with ischemic heart disease in both low non-optimal temperature environment **(C)**. The trend and prediction of DALYs in patients with ischemic heart disease in both low non-optimal temperature environments **(D)**.

To establish an evidence-based framework for IHD prevention and therapeutic interventions under extreme thermal conditions, we implemented an Age-Period-Cohort modeling approach to project future global disease burden trajectories of IHD across varying climatic temperature extremes, as depicted in the accompanying analytical figures. During the study period, 1990 to 2021, under high-temperature exposure conditions, the ASR for IHD mortality demonstrated a substantial increase of 48.2%, while DALYs had a 51.4% increase. Epidemiological modeling indicates that this ascending trajectory will stabilize, with mortality burden projected to experience a 51.2% increase by 2050 relative to the 2021 baseline values, culminating in rates of 2.241-fold higher compared to those in the 1990. Correspondingly, DALYs are projected to increase by 55.5%, reaching 2.355 times the 1990 reference value. In contrast, under low-temperature exposure conditions, the ASR for ischemic heart disease declined by 36.9%, with DALYs decreasing by 35.9% over the 1990–2021 observation period. Predictive modeling indicates that this favorable descending trajectory will persist, with mortality burden anticipated to achieve a 30.1% decrease by 2050, representing 44.0% reduction relative to the 1990 baseline value. Similarly, DALYs are projected to decline by 27.4%, constituting 46.4% reduction relative to the 1990 reference value (Figure 6).

## 4 Discussion

**Figure 7:**
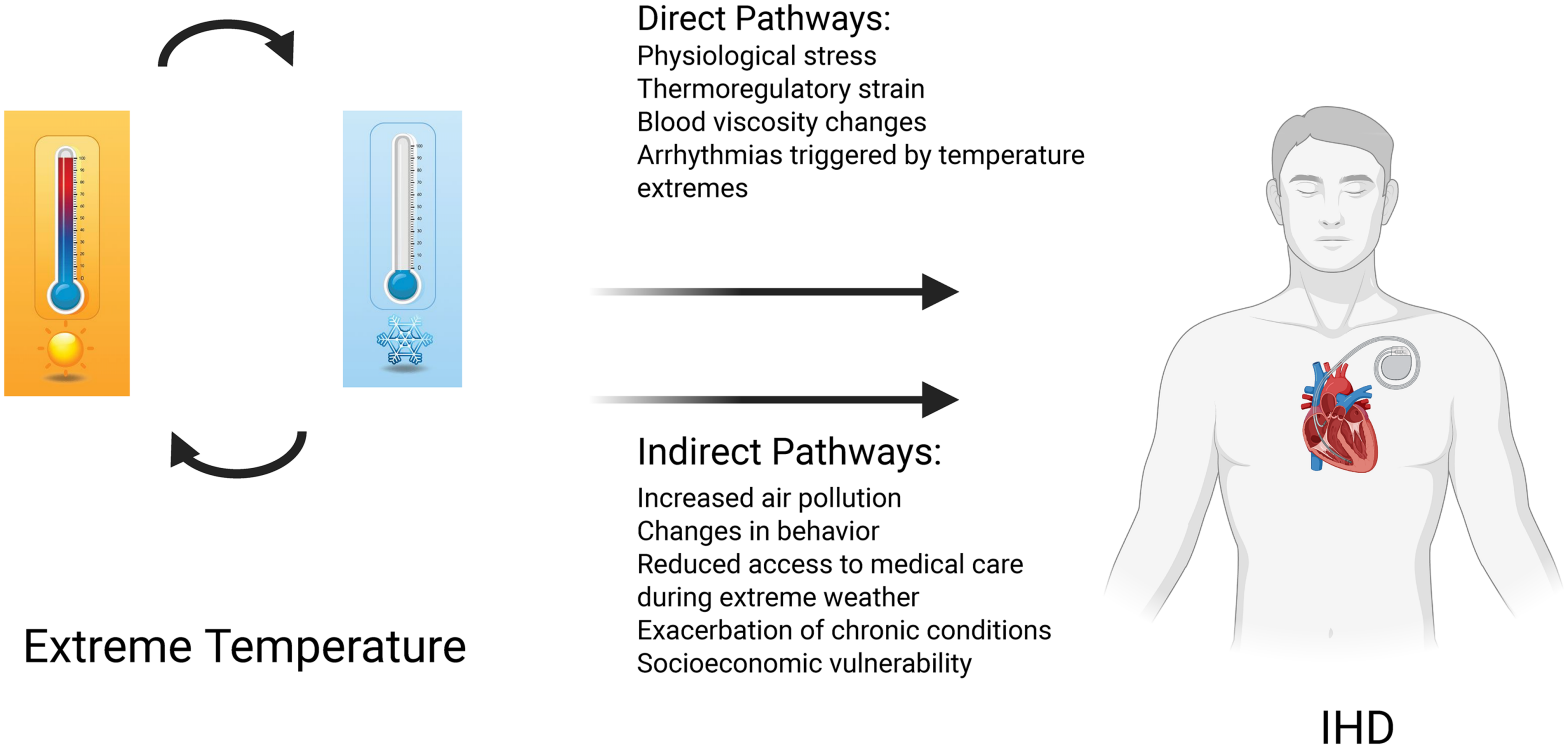
Conceptual model illustrating the direct and indirect pathways linking extreme temperature exposure to ischemic heart disease(IHD) outcomes.

This comprehensive epidemiological investigation reveals a significant increase in the global burden of heat-attributable IHD from 1990 to 2021, characterized by pronounced ASR increase among the advanced-age populations, males, and territories with low-to-middle SDI. Although ASR attributable to cold exposure demonstrated an overall declining trajectory, the absolute disease burden remains substantial, emphasizing the enduring public health challenge posed by extreme thermal exposures. The following analytical discourse contextualizes these key epidemiological findings through comprehensive data synthesis and integration of relevant literature (Figure 7).

### 4.1 Impact and temporal trends of high and low non-optimal temperatures on IHD burden

Comprehensive systematic analysis demonstrates that heat-attributable IHD mortality, DALYs, and ASR exhibited significant increase during the study period, with pronounced impacts arising from low-to-middle SDI territories. This epidemiological trajectory aligns with the patterns of anthropogenic climate change, intensified frequency of extreme thermal events, and the unequal distribution of healthcare resources. Demographic transition dynamics and epidemiological shifts further amplify regional heterogeneity in cardiovascular disease burden. Notably, male populations demonstrated significantly elevated mortality and morbidity burden—male-to-female ratio of 1.416—presumably attributable to enhanced occupational outdoor thermal exposure and inherent physiological limitation in thermoregulatory capacity compared to their female conuterparts (13, 14). Notably, older adult cohorts, individuals of ≥70 years, experienced the most pronounced increase in ASR, corroborating the findings from previous studies reporting the synergistic pathophysiological effects of thermal stress and age-associated cardiovascular comorbidities (15, 16).

We observed gender disparity in cold-attributable mortality (male-to-female ratio of 1.224:1), with significant correlation noted between elevated smoking prevalence and atherosclerotic disease burden among male populations (10). Furthermore, emerging pathophysiological hypotheses propose differential gender-specific thermal vulnerability patterns. These hypotheses indicate that females may exhibit enhanced physiological susceptibility to cold-induced cardiovascular stress, while males demonstrate greater vulnerability to high non-optimal temperature related thermal effects. Postmenopausal women constitute a particularly high-risk demographic, potentially exhibiting heightened vasoconstrictive responses to hypothermic exposure due to declining estrogen levels, thereby amplifying myocardial stress and elevating cardiovascular risk under hypothermic environmental conditions (17).

### 4.2 Regional heterogeneity and socioeconomic determinants

Jena et al. and concurrent epidemiological studies highlight substantial regional disparities in temperature-attributable mortality (18, 19), with low-to-middle SDI territories accounting for 75% of global high non-optimal temperature-related IHD fatalities. Structural healthcare inadequacies in South Asian and African regions—including limited access to air conditioning, heightened occupational exposure to thermal extremes, and pervasive socioeconomic deprivation—n significantly amplify population-level cardiovascular risk (20). Conversely, high-SDI regions such as Canada and Germany, achieved substantial annual reductions in cold-attributable ASR of 6.8% through enhanced infrastructure development and optimized emergency cardiovascular care systems.

Geographically, North African, Middle Eastern, and South Asian territories experience a convergent “triple risk” paradigm comprising extreme thermal exposure (21), outdoor occupational dependency, and healthcare system deficiencies, as exemplified by Iraq’s elevated ASR of 24.704 per 100,000 individuals. Concurrently, East Asian regions demonstrate predominant global cold-attributable DALY burden, with China accounting for 55.8% of the global burden, potentially correlated with exposure to winter-associated atmospheric pollution (22). India reported a substantial burden of 40,245 heat-attributable deaths, which is indicative of systemic deficiencies in energy accessibility and inadequate infrastructure to mitigate thermal impacts (23). Conversely, China’s comprehensive industrial and healthcare system reforms exhibits a projected reduction of 48.12% in DALY (24). However, China continues to experience epidemiological challenges due to demographic aging dynamics and persistent urban–rural healthcare disparities (25).

### 4.3 Future trend projections and public health implications

Age-Period-Cohort model projections demonstrate that the epidemiological burden of heat-attributable IHD will exhibit sustained increase, with mortality and DALYs projected to increase by 51.2 and 55.5%, respectively, by 2050 relative to the 2021 baseline values. Conversely, the burden of cold-attributable IHD is anticipated to exhibit progressive decline, with mortality and DALYs decreasing by 30.1 and 27.4% by 2050, corroborating findings reported by Yu et al (26). These epidemiological trajectories suggest that anthropogenic climate change will substantially exacerbate high non-optimal temperature-related cardiovascular health burdens. Additionally, reductions in low non-optimal temperature-related burdens potentially indicate the progressive advancements in medical technology and socioeconomic development. Nevertheless, substantial disparities persist across gender demographics, age stratification, and SDI regional classifications, underscoring the need for the development of targeted and differentiated public health intervention strategies. Accumulating epidemiological evidence validate the existence of significant associations between non-optimal temperature exposure and elevated risks of myocardial infarction, cerebrovascular accidents, and cardiovascular mortality (27). Specifically, population-based studies from Japan have shown that hypothermic conditions disproportionately increase hospitalization risks for cardiovascular diseases, with heart failure exhibiting a greater sensitivity compared to IHD and stroke (28). Proposed evidence-based mitigation strategies encompass the following: (1) implementation of integrated “high-temperature warning-occupational cessation compensation” linkage mechanisms during extreme meteorological events; (2) enhancement of community-based medical grid management systems—including systematic home visitation programs by primary care physicians—in demographically aging societies; (3) optimization of early-warning surveillance systems for high-risk population cohorts.

### 4.4 Policy recommendations

This investigation provides comprehensive systematic analysis of the impacts of global temperature extremes on IHD burden, thereby providing an evidence-based foundation for climate-adaptive public health policy frameworks. The escalating burden of heat-attributable IHD necessitates the development of enhanced institutional capacity to mitigate extreme thermal event, particularly in low-to-middle SDI territories. Notably, in such regions prioritizing equitable distribution of healthcare resources and development of climate resilience infrastructure constitutes a critical public health imperative. While declining cold-attributable burdens demonstrate the beneficial outcomes of medical technological advancement and socioeconomic development, sustained surveillance and protection of the older adult and vulnerable population cohorts in high-risk geographical areas remains a significant necessity. Evidence-based policy recommendations for the high-risk populations and regions include the following: (1) promoting deployment of comprehensive climate-adaptive infrastructure in high-exposure regions, encompassing thermally responsive architectural design standards and integrated heating or cooling distribution systems to mitigate temperature-related cardiovascular stress; (2) strengthening health monitoring protocols for advanced-age populations and individuals with chronic cardiovascular comorbidities, with the implementation of targeted protective interventions during extreme meteorological events through integrated early-warning and response mechanisms; (3) Implementing inclusive energy equity policies to guarantee that underserved communities in low- to middle-SDI regions maintain access to climate control and vital cardiovascular health services; (4) strengthening multilateral international cooperation frameworks to address the global threat of anthropogenic climate change to cardiovascular health outcomes, fostering knowledge transfer, resource sharing, and coordinated policy implementation across diverse socioeconomic contexts. These evidence-based recommendations provide a strategic framework for policymakers to develop comprehensive, equity-focused interventions that address the complex intersection of climate change and global cardiovascular health disparities.

### 4.5 Limitations and future directions

While this study offers a comprehensive perspective for the mitigation and prevention of IHD-related burden due to thermal extremes through the use of data from the GBD 2021 study and multidimensional analysis, it has several limitations that warrant acknowledgment. For instance, indirect climate-related impacts—such as air pollution and psychological stress—on IHD were not thoroughly explored. Furthermore, the confounding effects potentially due to regional disparities in climate adaptability and healthcare systems were not fully adjusted for, necessitating future research to include localized data for deeper insights.

## 5 Conclusion

This study highlights the profound impact of climate change on IHD burden and underscores the urgent need for coordinated global and regional climate action. Future investigations should focus on elucidating the mechanistic associations between climate change and other chronic diseases, while evaluating the efficacy of multilevel interventions to comprehensively and effectively resolve climate-related health challenges.

## Data Availability

The original contributions presented in the study are included in the article/Supplementary material, further inquiries can be directed to the corresponding author.
