# Supplementary material for: Diverging trends in the global burden of ischemic heart disease attributable to non-optimal temperatures: a historical analysis (1990–2021) and 2050 projections
Source: Front Public Health. 2025 Jul 30;13:1593346. doi: 10.3389/fpubh.2025.1593346 (PMC12343581; doi:10.3389/fpubh.2025.1593346)
Supplement: Supplementary file 1 [file Table_1.docx]

##BAPC预测#######

library(easyGBDR)

#版本配置

GBD_edition(edition = 2021)#2019/2021

setwd("C:/Users/14278/Desktop/Stroke/预测")

df1 <- GBDread(folder=T,foldername ="C:/Users/14278/Desktop/Stroke/预测")

#####Low temperature-Death########

#查看数据

bapc_results1 <- GBDbapc_prediction(

data = df1,#数据及以下参数填写要求均同Nordpred

measure_name = "Deaths",

cause_name = "Ischemic stroke",

location_name = "Global",

rei_name = "Low temperature",

By_sex = F,

predyear = 2050,

full_age_adjusted = T,

rate_lessen = NULL,

pop_predict = "WHO"

)

str(bapc_results1)

# 加载必要的包（如果还没安装，需要先安装）

library(ggsci) # NEJM配色

# 提取所需数据

number_data <- bapc_results1$all_age_projection

asr_data <- bapc_results1$ASR

# 合并数据并整理

combined_data <- rbind(

data.frame(

year = number_data$year,

value = number_data$pred_val,

type = "Number",

sex = number_data$sex,

lower = number_data$pred_low,

upper = number_data$pred_up

),

data.frame(

year = asr_data$year,

value = asr_data$pred_val,

type = "ASR",

sex = asr_data$sex,

lower = asr_data$pred_low,

upper = asr_data$pred_up

)

)

# 创建双轴图

p1 <- ggplot() +

# 添加柱状图 (Number)

geom_bar(data = subset(combined_data, type == "Number" & year <= 2036),

aes(x = year, y = value, fill = sex),

stat = "identity", position = "dodge", alpha = 0.8, width = 0.7) +

# 添加线图 (ASR)

geom_line(data = subset(combined_data, type == "ASR" & year <= 2036),

aes(x = year, y = value * max(subset(combined_data, type == "Number" & year <= 2036)$value) /

max(subset(combined_data, type == "ASR" & year <= 2036)$value),

color = sex, linetype = sex), size = 1) +

# 添加置信区间

geom_ribbon(data = subset(combined_data, type == "ASR" & year <= 2036),

aes(x = year,

ymin = lower * max(subset(combined_data, type == "Number" & year <= 2036)$value) /

max(subset(combined_data, type == "ASR" & year <= 2036)$value),

ymax = upper * max(subset(combined_data, type == "Number" & year <= 2036)$value) /

max(subset(combined_data, type == "ASR" & year <= 2036)$value),

fill = sex), alpha = 0.1) +

# 设置双Y轴

scale_y_continuous(

name = "Number",

labels = scales::comma, # 添加千位分隔符

sec.axis = sec_axis(

~ . * max(subset(combined_data, type == "ASR" & year <= 2036)$value) /

max(subset(combined_data, type == "Number" & year <= 2036)$value),

name = "Age-standardized rate (per 100,000)"

)

) +

# 使用NEJM配色

scale_fill_nejm() +

scale_color_nejm() +

# 设置x轴刻度

scale_x_continuous(breaks = seq(1990, 2036, by = 5)) +

# 添加主题和标签

theme_minimal() +

theme(

axis.title.y.left = element_text(color = "black", size = 12),

axis.title.y.right = element_text(color = "black", size = 12),

axis.text = element_text(size = 10),

legend.position = "bottom",

legend.title = element_text(size = 12),

legend.text = element_text(size = 10),

panel.grid.minor = element_blank(),

panel.grid.major.x = element_blank(),

panel.border = element_rect(fill = NA, color = "black", linewidth = 0.5),

plot.title = element_text(size = 14, face = "bold", hjust = 0.5),

plot.margin = margin(t = 20, r = 20, b = 20, l = 20)

) +

labs(

x = "Year",

title = "Death-Low temperature",

subtitle = " ",

fill = "Sex",

color = "Sex"

)

# 保存图片

ggsave("Deaths-Low temperature.pdf", p1, width = 8, height = 6, dpi = 300, device = cairo_pdf)

#####Low temperature-DALYs########

#查看数据

bapc_results2 <- GBDbapc_prediction(

data = df1,#数据及以下参数填写要求均同Nordpred

measure_name = "DALYs (Disability-Adjusted Life Years)",

cause_name = "Ischemic stroke",

location_name = "Global",

rei_name = "Low temperature",

By_sex = F,

predyear = 2050,

full_age_adjusted = T,

rate_lessen = NULL,

pop_predict = "WHO"

)

# 加载必要的包（如果还没安装，需要先安装）

library(ggsci) # NEJM配色

# 提取所需数据

number_data <- bapc_results2$all_age_projection

asr_data <- bapc_results2$ASR

# 合并数据并整理

combined_data <- rbind(

data.frame(

year = number_data$year,

value = number_data$pred_val,

type = "Number",

sex = number_data$sex,

lower = number_data$pred_low,

upper = number_data$pred_up

),

data.frame(

year = asr_data$year,

value = asr_data$pred_val,

type = "ASR",

sex = asr_data$sex,

lower = asr_data$pred_low,

upper = asr_data$pred_up

)

)

# 创建双轴图

p2 <- ggplot() +

# 添加柱状图 (Number)

geom_bar(data = subset(combined_data, type == "Number" & year <= 2036),

aes(x = year, y = value, fill = sex),

stat = "identity", position = "dodge", alpha = 0.8, width = 0.7) +

# 添加线图 (ASR)

geom_line(data = subset(combined_data, type == "ASR" & year <= 2036),

aes(x = year, y = value * max(subset(combined_data, type == "Number" & year <= 2036)$value) /

max(subset(combined_data, type == "ASR" & year <= 2036)$value),

color = sex, linetype = sex), size = 1) +

# 添加置信区间

geom_ribbon(data = subset(combined_data, type == "ASR" & year <= 2036),

aes(x = year,

ymin = lower * max(subset(combined_data, type == "Number" & year <= 2036)$value) /

max(subset(combined_data, type == "ASR" & year <= 2036)$value),

ymax = upper * max(subset(combined_data, type == "Number" & year <= 2036)$value) /

max(subset(combined_data, type == "ASR" & year <= 2036)$value),

fill = sex), alpha = 0.1) +

# 设置双Y轴

scale_y_continuous(

name = "Number",

labels = scales::comma, # 添加千位分隔符

sec.axis = sec_axis(

~ . * max(subset(combined_data, type == "ASR" & year <= 2036)$value) /

max(subset(combined_data, type == "Number" & year <= 2036)$value),

name = "Age-standardized rate (per 100,000)"

)

) +

# 使用NEJM配色

scale_fill_nejm() +

scale_color_nejm() +

# 设置x轴刻度

scale_x_continuous(breaks = seq(1990, 2036, by = 5)) +

# 添加主题和标签

theme_minimal() +

theme(

axis.title.y.left = element_text(color = "black", size = 12),

axis.title.y.right = element_text(color = "black", size = 12),

axis.text = element_text(size = 10),

legend.position = "bottom",

legend.title = element_text(size = 12),

legend.text = element_text(size = 10),

panel.grid.minor = element_blank(),

panel.grid.major.x = element_blank(),

panel.border = element_rect(fill = NA, color = "black", linewidth = 0.5),

plot.title = element_text(size = 14, face = "bold", hjust = 0.5),

plot.margin = margin(t = 20, r = 20, b = 20, l = 20)

) +

labs(

x = "Year",

title = "DALYs-Low temperature",

subtitle = " ",

fill = "Sex",

color = "Sex"

)

# 保存图片

ggsave("DALYs-Low temperature.pdf", p2, width = 8, height = 6, dpi = 300, device = cairo_pdf)

#####High temperature-Death########

#查看数据

bapc_results3 <- GBDbapc_prediction(

data = df1,#数据及以下参数填写要求均同Nordpred

measure_name = "Deaths",

cause_name = "Ischemic stroke",

location_name = "Global",

rei_name = "High temperature",

By_sex = F,

predyear = 2050,

full_age_adjusted = T,

rate_lessen = NULL,

pop_predict = "WHO"

)

library(ggsci) # NEJM配色

# 提取所需数据

number_data <- bapc_results3$all_age_projection

asr_data <- bapc_results3$ASR

# 合并数据并整理

combined_data <- rbind(

data.frame(

year = number_data$year,

value = number_data$pred_val,

type = "Number",

sex = number_data$sex,

lower = number_data$pred_low,

upper = number_data$pred_up

),

data.frame(

year = asr_data$year,

value = asr_data$pred_val,

type = "ASR",

sex = asr_data$sex,

lower = asr_data$pred_low,

upper = asr_data$pred_up

)

)

# 创建双轴图

p3 <- ggplot() +

# 添加柱状图 (Number)

geom_bar(data = subset(combined_data, type == "Number" & year <= 2036),

aes(x = year, y = value, fill = sex),

stat = "identity", position = "dodge", alpha = 0.8, width = 0.7) +

# 添加线图 (ASR)

geom_line(data = subset(combined_data, type == "ASR" & year <= 2036),

aes(x = year, y = value * max(subset(combined_data, type == "Number" & year <= 2036)$value) /

max(subset(combined_data, type == "ASR" & year <= 2036)$value),

color = sex, linetype = sex), size = 1) +

# 添加置信区间

geom_ribbon(data = subset(combined_data, type == "ASR" & year <= 2036),

aes(x = year,

ymin = lower * max(subset(combined_data, type == "Number" & year <= 2036)$value) /

max(subset(combined_data, type == "ASR" & year <= 2036)$value),

ymax = upper * max(subset(combined_data, type == "Number" & year <= 2036)$value) /

max(subset(combined_data, type == "ASR" & year <= 2036)$value),

fill = sex), alpha = 0.1) +

# 设置双Y轴

scale_y_continuous(

name = "Number",

labels = scales::comma, # 添加千位分隔符

sec.axis = sec_axis(

~ . * max(subset(combined_data, type == "ASR" & year <= 2036)$value) /

max(subset(combined_data, type == "Number" & year <= 2036)$value),

name = "Age-standardized rate (per 100,000)"

)

) +

# 使用NEJM配色

scale_fill_nejm() +

scale_color_nejm() +

# 设置x轴刻度

scale_x_continuous(breaks = seq(1990, 2036, by = 5)) +

# 添加主题和标签

theme_minimal() +

theme(

axis.title.y.left = element_text(color = "black", size = 12),

axis.title.y.right = element_text(color = "black", size = 12),

axis.text = element_text(size = 10),

legend.position = "bottom",

legend.title = element_text(size = 12),

legend.text = element_text(size = 10),

panel.grid.minor = element_blank(),

panel.grid.major.x = element_blank(),

panel.border = element_rect(fill = NA, color = "black", linewidth = 0.5),

plot.title = element_text(size = 14, face = "bold", hjust = 0.5),

plot.margin = margin(t = 20, r = 20, b = 20, l = 20)

) +

labs(

x = "Year",

title = "death-High temperature",

subtitle = " ",

fill = "Sex",

color = "Sex"

)

# 保存图片

ggsave("death-High temperature.pdf", p3, width = 8, height = 6, dpi = 300, device = cairo_pdf)

#####High temperature-DALYs########

#查看数据

bapc_results4 <- GBDbapc_prediction(

data = df1,#数据及以下参数填写要求均同Nordpred

measure_name = "DALYs (Disability-Adjusted Life Years)",

cause_name = "Ischemic stroke",

location_name = "Global",

rei_name = "High temperature",

By_sex = F,

predyear = 2050,

full_age_adjusted = T,

rate_lessen = NULL,

pop_predict = "WHO"

)

# 加载必要的包（如果还没安装，需要先安装）

library(ggsci) # NEJM配色

# 提取所需数据

number_data <- bapc_results4$all_age_projection

asr_data <- bapc_results4$ASR

# 合并数据并整理

combined_data <- rbind(

data.frame(

year = number_data$year,

value = number_data$pred_val,

type = "Number",

sex = number_data$sex,

lower = number_data$pred_low,

upper = number_data$pred_up

),

data.frame(

year = asr_data$year,

value = asr_data$pred_val,

type = "ASR",

sex = asr_data$sex,

lower = asr_data$pred_low,

upper = asr_data$pred_up

)

)

# 创建双轴图

p4 <- ggplot() +

# 添加柱状图 (Number)

geom_bar(data = subset(combined_data, type == "Number" & year <= 2036),

aes(x = year, y = value, fill = sex),

stat = "identity", position = "dodge", alpha = 0.8, width = 0.7) +

# 添加线图 (ASR)

geom_line(data = subset(combined_data, type == "ASR" & year <= 2036),

aes(x = year, y = value * max(subset(combined_data, type == "Number" & year <= 2036)$value) /

max(subset(combined_data, type == "ASR" & year <= 2036)$value),

color = sex, linetype = sex), size = 1) +

# 添加置信区间

geom_ribbon(data = subset(combined_data, type == "ASR" & year <= 2036),

aes(x = year,

ymin = lower * max(subset(combined_data, type == "Number" & year <= 2036)$value) /

max(subset(combined_data, type == "ASR" & year <= 2036)$value),

ymax = upper * max(subset(combined_data, type == "Number" & year <= 2036)$value) /

max(subset(combined_data, type == "ASR" & year <= 2036)$value),

fill = sex), alpha = 0.1) +

# 设置双Y轴

scale_y_continuous(

name = "Number",

labels = scales::comma, # 添加千位分隔符

sec.axis = sec_axis(

~ . * max(subset(combined_data, type == "ASR" & year <= 2036)$value) /

max(subset(combined_data, type == "Number" & year <= 2036)$value),

name = "Age-standardized rate (per 100,000)"

)

) +

# 使用NEJM配色

scale_fill_nejm() +

scale_color_nejm() +

# 设置x轴刻度

scale_x_continuous(breaks = seq(1990, 2036, by = 5)) +

# 添加主题和标签

theme_minimal() +

theme(

axis.title.y.left = element_text(color = "black", size = 12),

axis.title.y.right = element_text(color = "black", size = 12),

axis.text = element_text(size = 10),

legend.position = "bottom",

legend.title = element_text(size = 12),

legend.text = element_text(size = 10),

panel.grid.minor = element_blank(),

panel.grid.major.x = element_blank(),

panel.border = element_rect(fill = NA, color = "black", linewidth = 0.5),

plot.title = element_text(size = 14, face = "bold", hjust = 0.5),

plot.margin = margin(t = 20, r = 20, b = 20, l = 20)

) +

labs(

x = "Year",

title = "DALYs-High temperature",

subtitle = " ",

fill = "Sex",

color = "Sex"

)

# 保存图片

ggsave("DALYs-High temperature.pdf", p4, width = 8, height = 6, dpi = 300, device = cairo_pdf)

library(ggplot2)

library(ggsci)

library(cowplot)

library(dplyr)

library(patchwork)

# 合并图片并将legend放在底部

combined_plot <- (p1 + p2) / (p3 + p4) +

plot_layout(guides = "collect") &

theme(legend.position = "bottom")

# 保存图片

ggsave("combined_predictions.pdf",

combined_plot,

width = 300,

height = 250,

units = "mm",

dpi = 300)

str(bapc_results2)

# 为每个数据框创建CSV文件

write.csv(bapc_results1$all_age_projection,

"all_age_projection.csv",

row.names = FALSE)

write.csv(bapc_results1$crude_rate,

"crude_rate.csv",

row.names = FALSE)

write.csv(bapc_results1$ASR,

"ASR.csv",

row.names = FALSE)

write.csv(bapc_results1$Age_standardized_projection,

"Age_standardized_projection.csv",

row.names = FALSE)

write.csv(bapc_results1$age_specific_rate,

"age_specific_rate.csv",

row.names = FALSE)

write.csv(bapc_results1$age_specific_projection,

"age_specific_projection.csv",

row.names = FALSE)

str(bapc_results2)

write.csv(bapc_results2$all_age_projection,

"all_age_projection.csv",

row.names = FALSE)

write.csv(bapc_results2$crude_rate,

"crude_rate.csv",

row.names = FALSE)

write.csv(bapc_results2$ASR,

"ASR.csv",

row.names = FALSE)

write.csv(bapc_results2$Age_standardized_projection,

"Age_standardized_projection.csv",

row.names = FALSE)

write.csv(bapc_results2$age_specific_rate,

"age_specific_rate.csv",

row.names = FALSE)

write.csv(bapc_results2$age_specific_projection,

"age_specific_projection.csv",

row.names = FALSE)

str(bapc_results3)

write.csv(bapc_results3$all_age_projection,

"all_age_projection.csv",

row.names = FALSE)

write.csv(bapc_results3$crude_rate,

"crude_rate.csv",

row.names = FALSE)

write.csv(bapc_results3$ASR,

"ASR.csv",

row.names = FALSE)

write.csv(bapc_results3$Age_standardized_projection,

"Age_standardized_projection.csv",

row.names = FALSE)

write.csv(bapc_results3$age_specific_rate,

"age_specific_rate.csv",

row.names = FALSE)

write.csv(bapc_results3$age_specific_projection,

"age_specific_projection.csv",

row.names = FALSE)

str(bapc_results4)

write.csv(bapc_results4$all_age_projection,

"all_age_projection.csv",

row.names = FALSE)

write.csv(bapc_results4$crude_rate,

"crude_rate.csv",

row.names = FALSE)

write.csv(bapc_results4$ASR,

"ASR.csv",

row.names = FALSE)

write.csv(bapc_results4$Age_standardized_projection,

"Age_standardized_projection.csv",

row.names = FALSE)

write.csv(bapc_results4$age_specific_rate,

"age_specific_rate.csv",

row.names = FALSE)

write.csv(bapc_results4$age_specific_projection,

"age_specific_projection.csv",

row.names = FALSE)
